# Skin Manifestation of Human Monkeypox

**DOI:** 10.3390/jcm12030914

**Published:** 2023-01-24

**Authors:** Xue Wang, Wenhui Lun

**Affiliations:** Department of Dermatology and Venereology, Beijing Ditan Hospital Capital Medical University, Beijing 100015, China

**Keywords:** monkeypox, viral dermatosis, skin manifestations, HIV–MPX co-infection

## Abstract

Monkeypox is a zoonotic infectious disease caused by the monkeypox virus (MPXV) infection, which is mainly manifested as characteristic rashes. It spreads mainly through direct skin-to-skin contact. In some cases, respiratory transmission occurs through contact with respiratory droplets when in close contact with an infected person for a long time. The monkeypox outbreak in 2022 frequently occurred in the MSM (men who have sex with men) population, raising concerns about whether monkeypox could be transmitted through sexual behavior. This article mainly reviews the research progress of skin manifestations of monkeypox, including typical and atypical rashes of monkeypox, and different skin manifestations in some special groups, such as children, pregnant women and individuals co-infected with HIV (human immunodeficiency virus) and MPXV. At present, dermatologists are not very familiar with the diagnosis and treatment of monkeypox, it is therefore necessary to review the skin manifestations of monkeypox, which can help clinicians diagnose the sporadic cases and monitor the occurrence of monkeypox early, particularly in people at higher risk of infection. Early clinical diagnosis and treatment can largely avoid serious complications and death from monkeypox.

## 1. Clinical Manifestations of Monkeypox

Monkeypox is a zoonotic viral disease caused by monkeypox virus infection. The main clinical manifestations are fever, rash and lymphadenopathy. It is usually a self-limited disease, in most cases, the disease can be relieved within a few weeks [1]. Initially, monkeypox was mainly endemic in Central and West Africa. In 1970, the first human case of monkeypox was confirmed in the equatorial region of the Congo [2], and more than 400 cases have been found in Africa since then. In 2003, the first monkeypox outbreak outside Africa occurred in the United States [3]. A total of 81 cases were involved, of which 47 cases were attributed to contact with prairie dogs infected by rodents imported from Ghana. After that, Israel, Britain, Singapore and other countries have found monkeypox infection among travelers returning from Nigeria since 2018 [4]. Since 2022, human monkeypox has occurred in many non-endemic countries around the world. As of 27 September, monkeypox cases have been reported in 106 non-endemic countries and regions, with a total of 66,551 cases. Imported cases have also been reported in some Asian countries, such as China, South Korea, Japan, India, Thailand, and Singapore so on [5]. Most of the atypical monkeypox outbreaks in many countries were caused by persistent transmission between non-travelers, largely among men who have sex with men, which raises concerns about whether monkeypox can be transmitted through sexual behavior [1]. On 23 July 2022 the World Health Organization announced that the epidemic constituted a “public health emergency of international concern” [6]. At present, the research on the clinical manifestations of monkeypox is limited. This article mainly reviews the clinical manifestations of monkeypox.

Most of the early clinical data related to human monkeypox come from a follow-up survey of monkeypox outbreaks in Central and Western Africa in the 1980s. Human monkeypox is highly similar to smallpox in symptoms, severity and mortality, but the general symptoms are mild [7]. Smallpox vaccination can affect the severity of monkeypox, vaccinators tend to have fewer skin lesions, smaller skin lesions and are less likely to have serious complications [1]. The course of monkeypox infection can be divided into four stages: incubation period, prodromal stage, rash stage and crusting stage. Usually, the appearance of the rash represents the beginning of the infection period, and the lesions are contagious until the rash heals and all scabs fall off to form a layer of fresh skin [8].

### 1.1. Incubation Period

After the monkeypox virus enters the body from the mouth, nose, pharynx or skin damage, it replicates at the infected site, then spreads to the local lymph nodes and then forms the first viremia. Subsequently, the virus spreads to other tissues and organs, which is the incubation period of monkeypox [9]. Its incubation period is mostly 6–13 days, similar to smallpox, and is asymptomatic.

### 1.2. Prodromal Period

Its prodromal period lasts 1–4 days more [10], accompanied by fever (88%), headache (79%), itching (73%), enlarged lymph nodes (69%), myalgia (63%), sore throat (58%) and other prodromes [11]. There are also a few cases with atypical systemic symptoms or signs without obvious prodromal symptoms [12].

Fever is the most common prodromal symptom, in which the body temperature is usually between 101.3–104.9 °F, reaching the peak on the second day of fever [13]. Nearly half of the cases had a fever after the rash, and 27% of the patients still had a fever 2–3 days after the rash. At the same time, 37% of the patients had a secondary fever of 2–3 days during the rash and this clinical manifestation indicates the deterioration of the patient’s clinical condition [14]. In the study of monkeypox cases in Congo and the United States, fever was the main first symptom [13], but in the clinical review of monkeypox cases in Nigeria from 2017 to 2018, 65.7% of the patients presented with a rash, 34.3% with fever as the first symptom, and two cases with genital rash with ulcers as the first manifestation [15]. In their study, it was considered that patients with no obvious signs of fever may be caused by different routes of transmission. Previous studies have shown that the manners in which a person was exposed to MPXV, however, impacted both illness progression and symptom presentation, with complex exposures involving a combination of invasive (bite or scratch) and noninvasive contact found to be associated with a compressed incubation period [16]. The monkeypox outbreak in Nigeria was presumed to have been caused by human-to-human transmission of the main animal infectious source [17,18,19]. Monkeypox virus infection, which transmitted through some complex invasive exposure (such as scratches and bites), attenuated the obvious febrile premonitory characteristics of monkeypox.

Lymphadenopathy is a characteristic manifestation of monkeypox, which can be used as an important sign to distinguish monkeypox from varicella, smallpox and other infectious skin diseases [13,20]. According to statistics, more than 90% of monkeypox infections can develop lymphadenopathy, usually in the early stage of the disease, mostly 1–2 days after fever and occasionally 1–2 days after rash [14]. Enlarged lymph nodes up to 1–4 cm in diameter are hard, accompanied by local tenderness and pain. It often occurs in the neck, groin, retro auricular, submandibular or axillary lymph nodes, and can also spread throughout the body or any combination of these lymph nodes [21,22]. The presence of lymphadenopathy may indicate that the human immune system has a more effective immune recognition and response to monkeypox infection than smallpox infection, but this hypothesis still needs to be confirmed by further research.

### 1.3. Rash Period

**Figure 1:**
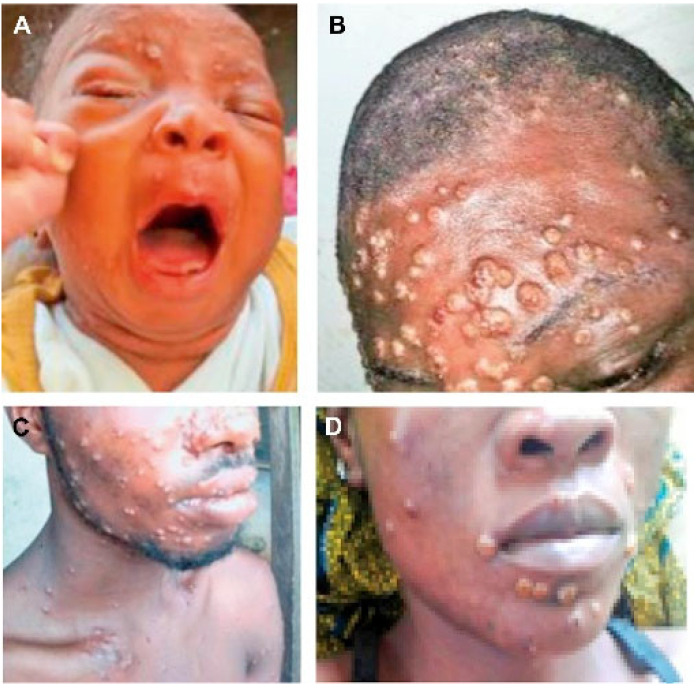
(**A**–**D**) Maculo-papular-vesicular-pustular monkeypox skin lesions of varying sizes on the face. (Courtesy of Nigeria Center for Disease Control, Abuja, Nigeria.) Reprinted with permission from Ref. [33]. Copyright 2019 the Infectious Disease Clinics of North America.

The rash usually occurs within 1–3 days after fever, and a small number of patients have rash and fever at the same time or more than 3 days after fever. The most common sites of skin lesions are face (97.5%), torso (92.5%), arms (87.5%), legs (85%), genitals (67.5%), scalp (62.5%), palms (55%), soles of feet (50%), mouth (37.5%) and eyes (25%) [15]. At present, there is no definitive evaluation for scalp involvement, but scalp involvement may be found based on available clinical case reports [19]. Rashes on the face and limbs were more common in early African outbreaks than on the torso, however, in this 2022 outbreak, skin lesions around the genitals (penis, testicles, labia and vagina) or on or near the anus were more common. In Nigeria, compared with previous studies in Congo and the United States, about 68% of patients in the former had genital lesions, while only about 3% of patients in the latter had genital lesions [13,23]. Cases of isolated genital, perianal or oral rash as the only skin lesions have also been reported [24]. Rashes usually first appear on the face (Figure 1), gradually spread to the limbs, torso and other parts, through mostly eccentric distribution [22,25], but a few can also be like chicken pox’s concentric distribution [26,27]. The rash begins with a red plaque of 2–5 mm (lesions with a flat base) (Figure 1A) that evolves into papules (hard lesions with a slight bulge) (Figure 1B), gradually to vesicles (lesions filled with transparent fluid) (Figure 1C) and then to pustules (lesions filled with yellowish fluid) (Figure 1D). Vesicles and pustules are mostly spherical, ranging from 0.5–2 cm in diameter, with a hard texture, deep involvement and clear boundaries. The central depression is umbilical fossa-like, which can be accompanied by obvious itching and pain. The number of lesions varies from several to thousands, and the severity of HMPX (Human monkeypox) can be divided into four grades according to the number of rashes: 1–25 as mild, 26–100 as moderate, 101–250 as grave and more than 250 as plus grave [28]. The development of the rash is slow, and the lesions in the same site are usually at the same stage of development, each stage lasts 1–2 days and the pustular period can last 5–7 days [29]. In this epidemic, there are also cases in which different stages of the rash occur at the same time [30,31,32].

### 1.4. Crusting Period

**Figure 2:**
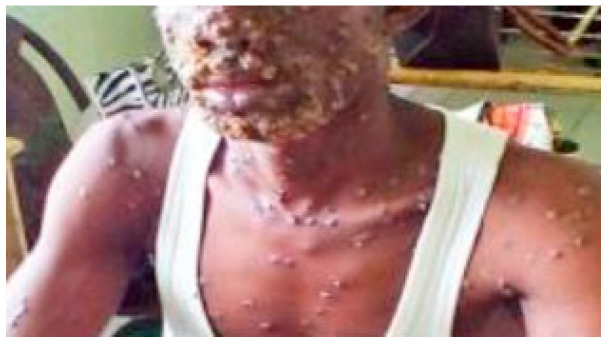
Extensive papulo-pustular monkeypox rashes with crust and scar formation. (Courtesy of Nigeria Center for Disease Control, Abuja, Nigeria.) Reprinted with permission from Ref. [33]. Copyright 2019 the Infectious Disease Clinics of North America.

From the onset to the scab shedding is about 2–4 weeks; the longest is 8 weeks [15]. The detached scab may be much smaller than the original lesion, erythema or pigmentation (Figure 2) may be left after the scab falls off and more than half of the scar remains. The scar can last for several years [1]. Usually, the appearance of a rash marks the beginning of the infection period [14,34,35] and is contagious until the crusts fall off. However, the Centers for Disease Control and Prevention (CDC) says prodromal periods can also be contagious [8]. Ferré VM et al. published the results of the study and found that some asymptomatic male sex samples tested positive for monkeypox virus through the Polymerase Chain Reaction (PCR), indicating that monkeypox may have asymptomatically transmistted [36].

## 2. Clinical Manifestations of Atypical Monkeypox

The incubation period of some cases was shorter, with an average of 8.5 days [37], and most of them had a history of sexual contact, such as anal and oral intercourse, before onset. The prodromal symptoms, such as fever and headache, were mild, and the local rash at the contact site was the first symptom [38,39]. The number of skin lesions is less than 20, and the distribution of skin lesions is more limited, mainly around the genitals and anus (78%), around the mouth and perioral mucosa (43%). During the rash period, the lesions may spread to distant areas such as the face, limbs and torso, showing skin damage at a different stage of development from the initial local rash. Localized lymph node enlargement was seen in 85% of the patients with skin lesions, but no extensive lymph node enlargement was observed. Some patients (91% have a history of anal intercourse) may have rectal pain, severe rectal tenesmus, exudation of purulent secretions, etc., while some patients (95% have a history of oral sex) have sore throat, dysphagia, swollen tonsils, ulcers on the surface and penile edema in a small number of patients. Most patients present other systems involvement (such as perianal pain, proctitis and tonsillitis) before the appearance of the rash (prodromal phase) or shortly after the rash (early clinical stage). Common complications requiring treatment in patients with atypical monkeypox are proctitis (25%), tonsillitis (10%), penile edema (8%), local abscess (3%), etc. [32]. Studies have shown that skin lesions at sexual contact sites suggest the possibility of sexual transmission [40,41]. The causes of these symptoms can be attributed to the invasive period of the disease. One explanation is that anal intercourse may destroy epithelial cells, causing the virus to enter the bloodstream through skin lesions at the inoculation site, which in turn affects adjacent organs, resulting in viremia in the early stages of local lesions; another explanation is that rectal mucosal lesions may have been omitted in the early stages of the patient. A similar phenomenon has been observed in patients with syphilis, that is, primary syphilis is rare in MSM (men who have sex with men), because syphilitic chancre in the rectum is more likely to be ignored. Emerging research evidence suggests that the monkeypox virus may be found in semen and other body fluids [24], but questions remain as to whether monkeypox can be transmitted sexually through semen and vaginal secretions. However, the extended definition of sexually transmitted diseases, such as syphilis and herpes simplex, includes pathogens in suppurative lesions on the surface of the genitals that can be transmitted through abrasions on the surface of the skin and mucosa [42]. The degree of keratosis of epithelial cells in anorectal and genitalia is low, the frequency of dendritic cells and antigen-presenting cells (such as macrophages) is high and infection is most likely to be obtained through sexual contact [43]. At the same time, some atypical monkeypox cases with syphilis, HIV, gonorrhea and chlamydia trachomatis infection and other transmitted diseases are also worthy of attention.

## 3. Complications

In addition to monkeypox’s characteristic rash, patients with monkeypox infection may also have other clinical manifestations such as secondary skin or soft tissue infection (19%), pneumonia (12%) and eye complications (4–5%) [44]. Serious complications, such as encephalitis, acute renal injury, myocarditis [30], hepatomegaly and scrotal edema [15], can lead to poor prognosis. Secondary skin infection is the most common complication of monkeypox. Extensive skin damage can also cause secondary bacterial infection [45], resulting in a large area of skin scar, which is the most common sequela that monkeypox can cause [14]. Pain caused by monkeypox is also a common reason of hospitalization. In the retrospective study by Ogoina D et al., 61 patients had anorectal mucosal involvement, and 21 patients had severe anorectal pain and needed to be hospitalized for analgesia [15,30]. If rashes occur around the genitals, anus and bilateral inguinal areas, it is prone to the appearance and secondary bacterial cellulitis [46]. Some studies have shown that septicemia or sepsis can occur when the number of skin lesions is more than 4500 [19], and can cause death of patients. In addition to skin, MPXV (monkeypox virus) can also cause damage to other mucous membranes. The oral mucosa involvement can lead to difficulty eating or drinking, and eventually oral ulcer, pharyngitis, tonsillitis and epiglottis. Oropharyngeal infection can also cause bronchopneumonia and lung distress, often occuring in the later stage of the disease, suggesting the secondary bacterial infection of the lung [26,47]. Periocular involvement can lead to corneal ulcers, conjunctivitis and blepharitis, and even permanent vision loss [48]. There are also patients who develop severe vomiting or diarrhea in the second week of infection with monkeypox, which can lead to severe dehydration [47].

## 4. Skin Histopathology

**Figure 3:**
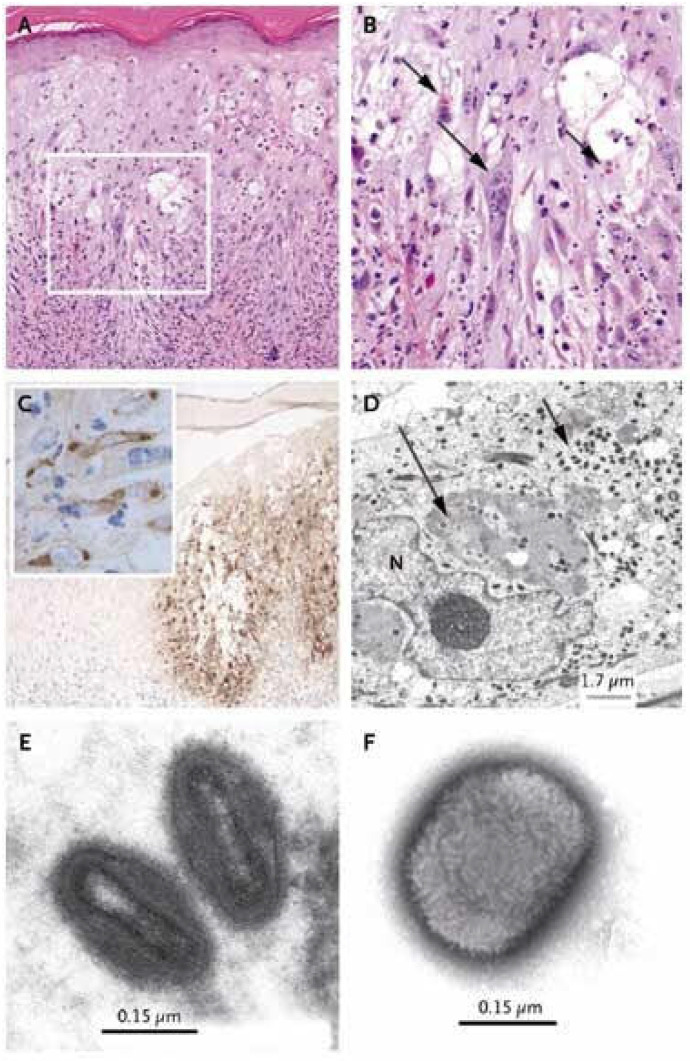
Histologic, Immunohistochemical and Ultrastructural Evaluation of the Skin-Biopsy Specimen from Patient. Panel (**A**) shows scattered degenerating and necrotic keratinocytes within the epidermis and a moderate inflammatory cell infiltrate within the epidermis and superficial dermis (hematoxylin and eosin, ×50). Panel (**B**) shows the boxed area in Panel A at a higher magnification (×200); a multinucleated cell (long arrow) and eosinophilic viral inclusion bodies (short arrows) are evident. Panel (**C**) shows immunohistochemical staining of orthopoxviruses antigen within the epidermis (horseradish peroxidase with hematoxylin counterstain, ×40). The inset shows immunoreactivity within individual keratinocytes (×250). Panel (**D**) shows virions within the cytoplasm of a keratinocyte and includes immature forms that are being assembled (long arrow) and clusters of mature virions (short arrow). N denotes nucleus. Panel (**E**) shows virions with dumbbell-shaped cores characteristic of poxviruses. Panel (**F**) shows a negatively stained virion from cell culture (phosphotungstic acid). The brick-shaped particle has regularly spaced, thread-like ridges on the exposed surface. Reprinted with permission from Ref. [3]. Copyright 2022 The New England Journal of Medicine.

The pathology of monkeypox is the same as that of other pox viruses, which is characterized by obvious eosinophilic inclusion bodies in the cytoplasm of epithelial cells. Papular lesions may include obvious thickening of the spinous layer, necrosis and proliferation of keratinocytes, basal vacuolation and lymphocyte infiltration around superficial and deep blood vessels of the dermis. Herpetic lesions or ulcers can be characterized by spongiform degeneration with reticular and balloon degeneration, infiltration of a large number of neutrophils, eosinophils and multinucleated giant cells, vasculitis can be observed in the superficial dermis and virus inclusion bodies appear in keratinocytes (Figure 3A,B). At the same time, virus antigens can also be detected in diseased epidermis, keratinocytes and dermal monocytes by specific immunohistochemistry (Figure 3C). Under an electron microscope, the cytoplasm of infected epidermal cells contains abundant immature and mature positive pox virus particles (Figure 3D) [33,34].

## 5. Differential Diagnosis

The World Health Organization lists smallpox, chickenpox, measles, bacterial dermatosis, drug eruption and syphilis as diseases to be differentiated from monkeypox [32]. Smallpox and severe chicken pox are the most confusing diseases of monkeypox. The clinical symptoms of monkeypox and smallpox are very similar, and the clinical manifestations of monkeypox and smallpox are difficult to distinguish because of the similar prodromal period, eruption process and the distribution of a rash. Therefore, the enlarged lymph nodes peculiar to monkeypox is a key feature to distinguish between the two. Monkeypox is generally less severe than smallpox in terms of complications morbidity, mortality, scab degree.

Severe cases of varicella are often misdiagnosed as monkeypox, except for the characteristic enlarged lymph nodes of monkeypox to distinguish the two, varicella usually has no obvious signs of fever, and the temperature during fever is usually lower than 38.8 °C. The skin lesions are centripetal, the lesions are smaller, involve the superficial surface and rarely involve the palms and soles of the feet, and the rashes on the torso are denser than those on the extremities and face. The progress of varicella lesions is faster than monkeypox: the former takes 3–5 days, while the latter takes an average of 12 days [49].

Other vaccinia virus infections are also easily confused with monkeypox [50]. Because the symptoms and signs of monkeypox are not specific, difficult to identify cases should be definitively diagnosed by laboratory testing.

## 6. Others

According to previous studies, monkeypox is more prone to severe complications and higher mortality than adults in children, pregnant women, and people with immunodeficiency (HIV-infected, transplant and cancer patients and genetic-based immunodeficiency patients) [51].

### 6.1. Children

In the early epidemic in Africa, children have been the main group of people with the disease. In 1970–1989, the median age of monkeypox cases was mostly 4–5 years old, and more than 80% of the cases were under 15 years old. In 2000–2019, the median age of monkeypox cases rose to 10–21 years old [52,53]. In the epidemic reported in Nigeria in 2017–2018, the median age of confirmed and suspected cases was 29 years (2 days–50 years) [18]. In 2022, monkeypox cases in non-endemic countries mainly occurred in adults [52]. As of 2 August 2022 the World Health Organization reported that among monkeypox patients with age information, 96 (0.6%) were under the age of 18, of which 42% needed to be hospitalized due to isolation or treatment, and there were at that time no child deaths [54].

According to epidemiological data, patients who have not been vaccinated with monkeypox vaccine are more likely to be transmitted through family environmental contact, and the infection rate can reach 12.3%, especially in children under the age of 15 [55]. In the early stage of Congo outbreak, more than 90% of the patients were children under the age of 15, and all deaths occurred in children aged 3 months to 8 years. The mortality rate ranged from 1.5% to 17%, and serious complications, such as septicemia, severe dehydration, blindness, pneumonia, encephalitis, etc. were more likely to occur [19,47,55]. Even in developed countries, children are more likely to have serious complications. In the epidemic in the United States in 2003, serious complications occurred in children with ICU, such as corneal ulcer, encephalitis and retropharyngeal abscess. The hospitalization rate of children was significantly higher than that of adults. There were sixteen cases (0.3%) of patients under 18 years old in the Spanish epidemic, of which one case had bacterial superinfection and required abscess drainage [54]. At present, although the risk of monkeypox transmission to children appears to be low, with the increase in adult cases, children will inevitably be exposed to the family, school or other similar environments, and can even be infected through sexual contact. Newborns may be infected with monkeypox virus during prenatal or perinatal contact with the mother [56].

### 6.2. Pregnant Women

The decline of the body’s immune system during pregnancy makes pregnant women also susceptible to monkeypox. There are few records of monkeypox infection among pregnant women in early African outbreaks, and limited attention has been paid to the changes in pregnancy outcomes after monkeypox infection and the impact of vertical transmission of monkeypox virus on fetuses. According to report by Mbala PK et al., four pregnant women with monkeypox virus during 6–18 weeks of pregnancy were followed up by Kohl General Hospital in Democratic Republic of the Congo from 2007 to 2011, of which two pregnant women infected with monkeypox had spontaneous abortions in the first trimester (the abortion product was not tested for monkeypox virus) and one pregnant woman with mild monkeypox gave birth to a full-term healthy child. The last pregnant woman was infected with moderate monkeypox at 18 weeks of gestation; after 3 weeks of fever, the virus load increased rapidly, the fetal heart stopped moving and the patient delivered a dead fetus with diffuse skin macular papules all over the fetal body involving the head, trunk and limbs, including palms and feet. The fetus developed severe liver involvement, including ascites and obvious hepatosplenomegaly, accompanied by increased vascular permeability. There was extensive autolysis after death, and no congenital malformation was found, which was consistent with fetal intrauterine death [57]. It is estimated that the risk of miscarriage in early pregnancy is 25–30% [58]. A sharp increase in monkeypox virus load may lead to the release of inflammatory cytokines in the placenta, resulting in cell damage. After testing, the dead fetus had the characteristics of monkeypox infection, in which virological, histological and serological evidence showed that there was vertical transmission. In addition, previous case reports of smallpox and other vaccinia viruses have shown that pregnant women have higher morbidity, abortion, premature and stillbirth rates, as well as a higher risk of hemorrhagic smallpox and mortality [57]. Consider that like smallpox infection [59], pregnant women infected with monkeypox virus are more serious than those who are not pregnant, especially during the late pregnancy. Although there is little information from clinical studies on the effects of monkeypox infection on pregnant women, vertical transmission of monkeypox is can be found to be associated with fetal death and congenital infection.

### 6.3. Monkeypox Complicated with HIV Infection

Previous studies on monkeypox in Nigeria did not identify HIV as an important cofactor in the epidemiology and clinical manifestations of monkeypox [60]. Only three cases of combined infection were found in suspected cases of monkeypox infection in Congo from 1996 to 1998 [61], and no combined infection was found in the epidemic in the United States in 2003 [3,19]. In a retrospective study of the epidemic situation in Nigeria from 2017 to 2018, nine patients with monkeypox were found to be co-infected with HIV [15]. The researchers found that, compared with HIV negative patients, the diameter of skin lesions in combined infection was more than 2 cm and the number of skin lesions was more than 100. Most of the skin lesions were semi-fused, almost all showed genital and perianal skin lesions, the bacterial superinfection rate of genital ulcers was higher and the hospital stay was more than two weeks. The course of disease was more than 28 days, the possibility of serious complications was higher and the mortality rate was higher than that of ordinary monkeypox patients. It seems that the existence of HIV-related immunosuppression has changed the history and course of monkeypox infection in people infected with MPXV.

In the monkeypox epidemic that occurred in 2022, most of the 96 confirmed cases in Portugal were men with HIV infection, which was mainly characterized by fever, local rash and inguinal lymphadenopathy [62]. The skin lesions first appeared in the genitals and perianal areas and then spread to the mouth, trunk, face and other parts [33,62]. Cohen MS et al. speculated that the appearance of dense skin lesions around the anus may be related to local inoculation of monkeypox virus, and the appearance of dense skin lesions aggravated by immune system dysfunction in acute HIV infection [63]. However, Thornhill, J.P et al. indicated that 41% of people infected with monkeypox virus were infected with HIV during the onset of the disease and 95% of them were receiving antiretroviral therapy. They did not considered that there to be significant difference in the clinical manifestation and severity of monkeypox with or without HIV infection. HIV infected patients were effectively controlled in their study [47].

It is worth noting that concomitant infections of MPXV and other sexually transmitted infections (STI) may also significantly increase the risk of HIV infection. Numerous cases of monkeypox have recently been reported with skin lesions in the genital area, which has been observed in cases of MSM infected with MPXV. It is worth noting that all MSM cases diagnosed with monkeypox have previous sexually transmitted infections, such as syphilis or HIV infection [21]. Previous studies have shown that 10% of HIV infections can be attributed to other sexually transmitted diseases such as chlamydia and gonorrhea [64]. Mathematical models that exploring the relationship between HIV and MPXV co-infection suggest that HIV may promote MPXV transmission, and vice versa [65]. At the same time, HIV positive patients should also pay attention to smallpox vaccination. Since the AIDS epidemic came after smallpox eradication stopped using the replicative common smallpox vaccine, it is not clear whether AIDS patients will have complications after receiving the replicative vaccine. Smallpox vaccination in HIV-positive people remains rare. According to available records, people who have been infected with HIV but have not been diagnosed with AIDS (CD4+ T lymphocytes > 200/mL) have no complications, whereas people with AIDS can develop disseminated vaccinia infection after vaccination [51]. Therefore, as the epidemic progresses, the focusing on educating these people about the complications of live viral vaccination and the probability of monkeypox infection will be the focus of public advocacy.

## 7. Conclusions

Over the past 50 years, the number of confirmed and suspected cases of human monkeypox has been increasing year by year globally. With the routine smallpox vaccination phased out since 1981, people under age of 40 years old lack adequate immunity to monkeypox virus. In the context of the current COVID-19 global pandemic, the outbreak of monkeypox virus is still deserves our attention.

At present, monkeypox is suspected to be a sexually transmitted disease in men who have sex with men, but it is unclear whether MPXV can be sexually transmitted, which needs to be supported by more research data. Clinicians need prompt detection and surveillance of possible monkeypox cases, particularly for this outbreak of monkeypox in non-endemic countries. Any people of high-risk population with blisters or pustular rashes (including those limited to genital or perianal areas) and lymphadenopathy must be screened timely, and common confounding diseases such as varicella, herpes simplex, herpes zoster and syphilis need to be excluded.

Compared with the high incidence rate and mortality rate of serious complications in African cases, the clinical prognosis of monkeypox cases in developed countries is better than that of the former. This phenomenon is most likely due to the differences in the medical environment. Without appropriate medical intervention, serious complications can also lead to death. Until more evidence is available, children, newborns, pregnant women, as well as immunocompromised patients, should be considered at high risk of monkeypox-related complications and deaths [19,66], and a high degree of vigilance is warranted.

Recent research evidence also highlights the link between monkeypox and sexually transmitted diseases such as AIDS [67]. Further research is needed, particularly in the area of whether HIV and monkeypox virus co-infection. With the widespread vaccination of the monkeypox vaccine, it is necessary to pay attention to the adverse effects and infection probability of high-risk groups, especially in those with insufficient immunity after live virus vaccination. Finally, we need to make efforts to control the global spread of monkeypox through case tracking, quarantine and vaccination. Whether monkeypox is a new sexually transmitted disease or not, we need to have a clear understanding of how monkeypox virus is transmitted. At present, more and more monkeypox cases are being reported around the world, so we need to perform more efforts to prevent it from spreading before it affects more people.

## Data Availability

Not applicable.
